# Porrigo (“Texas Mange”) in Horses

**Published:** 1891-09

**Authors:** Gerald E. Griffin

**Affiliations:** Veterinarian, Fifth U. S. Cavalry


### PORRIGO ("TEXAS MANGE") IN HORSES.

By Gerald E. Griffin, D. V. S.
Veterinarian, Fifth U. S. Cavalry.

Porrigo, or—as it is commonly known south of Kansas and Missouri—"Texas mange;" though simple in its nature is one of the most annoying diseases or conditions of the skin met with in southern latitudes, it is prevalent among all classes of horses (and frequently cows), particularly those confined to the stable, as are the horses in the cavalry service. The disease is observed almost invariably during the warmer months and disappears on the advent of cold, although in two instances it has been seen to afflict the patients all through the winter.

The first intimation we receive of the appearance of the disease amongst cavalry horses is a report from the Farriers (troop Vet'y. nurses) or troop commanders, that a certain animal in his troop is afflicted with "the mange," an examination is made, when the animal is found to be in apparently good health and all functions normal, the hairs about the superior portion of the tail or about the middle of the mane may appear rough and tumbled, and if the animal is watched for a short time he will be observed to back up against the heel-post (if in his stall), and rub his tail against this object in a most vigorous manner, while at the same time he appears to derive considerable satisfaction from the operation; if the animal chances to be on the picket-line, or turned loose in the corral he will endeavor to get his neck immediately under the picket-line, or through the fence, and there rub his mane most determinedly for various lengths of time, grunting with satisfaction the while, pulling the hairs out by the root and after a time leaving the skin of the parts rubbed, in an abraided condition which though undoubtedly painful, does not deter the animal from continuing the rubbing operation at the next opportunity; after a period of about two weeks the inclination to rub or scratch is so pronounced that patients have been observed, when tied to a ring above their heads and out of reach of surrounding fixtures, to use their hind feet wherewith to scratch the mane, and so expert did one of these animals become in this mode of scratching that he rubbed nearly all the hairs loose from within four inches of his withers to his foretop, before he was detected. Although the constitution of the patient remains unimpaired and his vigor undiminished, nevertheless, in a short time he presents a most deplorable appearance, the caudal appendage and neck being often denuded entirely of hair, he becomes an object of hatred to his rider and of ridicule to the remainder of the troopers, he still continues to rub and scratch the abraided and raw-looking parts until cold weather sets in when the itchiness seems to be relieved and the new hairs begin to make their appearance. In some cases the rubbing or scratching continues until the hair is rubbed off the back in patches up as far as the first lumbar vertebra and extending for a space of several inches on both sides of the median line, and anteriorly extending as far back as the tenth dorsal vertebra, giving the animal a most peculiar moth-eaten appearance.

On close examination of several cases that were held without treatment to observe the progress of the disease, it was found that it commenced with what appeared to be a small eruption—if it could be so called—of an apparently fluid nature, which in the course of 24 to 36 hours formed laminated scales, resembling fine white bran, this was accompanied by the itchiness and rubbing referred to above, and although the horse-brush was used several times a day these scales or exfoliations, continued to come off in undiminished numbers, while the skin and hair of the afflicted part presented a dirty, dusty appearance, as if it had been oiled and allowed to accumulate dust, and although the tail and mane were frequently washed, they again assumed this dusty appearance after a lapse of one or two days.

Is this disease contagious? it is thought not, as several experiments have been made, and long continued, all with negative results; when the first case was reported it was thought the rubbing was due to rectal irritation, and the animal was treated accordingly, but without success, when it was decided that the disease was "Texas mange," afflicted animals were tied in the same stalls, with those unaffected, to test its contagiousness, there was no other alternative, at all events, even if it was contagious, as the army, along with refusing to furnish a sufficient variety of drugs for veterinary purposes, have never, as far as known, made any attempt to furnish a veterinary hospital or an excuse for one although there is an order somewhere which says there *may* be a building set aside for this purpose, so that in suspicious cases or in cases of contagious diseases, the best animals in a troop are liable to destruction, by means of a bullet, for the reason that there is no safe place in which they may be held for observation.

Veterinary medicine will certainly never be much enlightened by the War Department from present appearances, but the results were negative, each troop at this post had from two to four animals with this disease, but in two years it has not extended beyond these particular animals, and it reappears regularly in these, year after year, when the hot weather sets in; exfoliations from affected animals were rubbed into the hair of the mane and tail of those who were free from the disease, but without producing any symptoms of the malady; as there was no microscope available, it was impossible to determine whether the symptoms were due to an organism or not, so the question still remains: Is this disease a dandruff, and if not, is it due to the presence of an organism, and if so, why does it not affect all parts of the body?

### Treatment.

As the number of drugs allowed by the Quartermaster-General, who furnishes the veterinary supplies, is very limited indeed, it did not take long to exhaust, the search for suitable remedies from this source and of course without affecting a cure. Drugs were purchased from private funds—it would be a waste of time to ask the Quartermaster's Department to furnish anything out of the old rut (as far as veterinary supplies are concerned) which is at least fifty years' behind the times—and after numerous experiments the following treatment was found to be effectual. Phytolacca extract, applied locally, after thoroughly washing the affected parts, the treatment to be continued every second day. The mercurial ointments and sub-caustics were tried with but poor results, but the treatment which gave the best satisfaction, while at the same time being cheap was as follows: equal parts of cosmoline and coal tar, heated to 200°F., to which was added powdered cantharidies, in the proportion of one to ten; this compound was applied every third day, when the rubbing ceased, the skin regained its tone, and in the course of three or four weeks a cure was in all cases effected.

It was stated by a Texas cattleman, who was well acquainted with "Texas mange," that he always affected a cure in his own animals by a liberal use of wagon grease, thinned down with coal oil, which was applied frequently with friction; this rather homely treatment was tried and with good results, but it was found that its virtue lay in the rancid wagon grease, which acted as a light blister, stimulating the skin and the growth of the hair. Another, and more elegant preparation, but rather expensive, which was found to be very effectual, was glycerine and tannin, eighty grains of the latter to one ounce of the former, applied with a sponge daily for one week, followed by embrocations of carbolized oil, if the surface is at all abraided.

As it is not definitely known to what to attribute the cause of this disease, the writer will be pleased to furnish scrapings from mane and tails of affected animals to any microscopist who may be interested enough in the above to make an investigation.

